# Mice with pR927Q and pI712M *Erbb4* Mutations Develop Features of Frontotemporal Dementia and ALS

**DOI:** 10.1002/alz70855_106736

**Published:** 2025-12-24

**Authors:** Demetri Spyropoulos, Dorea P Jenkins, Steven L Carroll

**Affiliations:** ^1^ Medical University of South Carolina, Charleston, SC, USA

## Abstract

**Background:**

Frontotemporal dementia (FTD) involves progressive deterioration of behavior, executive function, personality/traits and is evident structurally as frontotemporal lobar degeneration (FTLD). FTLD with pathological TDP43 neural/glial inclusions (FTLD‐TDP), often coexists with TDP43‐positive amyotrophic lateral sclerosis (ALS). Several families have been identified that inherit autosomal dominant FTLD/ALS or ALS alone and mutations in the gene encoding the *ERBB4* receptor tyrosine kinase (RTK). Although the neuropathology associated with *ERBB4* mutations remains obscure, *ERBB4* with p.R927Q or p.I712M mutations show reduced phosphorylation when stimulated with the ERBB4 ligand neuregulin‐1β, suggesting that reduced ERBB4 activity causes FTLD/ALS or ALS.

**Method:**

We generated mice carrying p.I712M (familial FTLD/ALS) or p.R927Q (familial ALS only) *Erbb4* mutations. We performed initial studies of behavior (Barnes Maze), gait‐mobility (Catwalk‐XT) and body composition (DXA scans) on *Erbb4* heterozygous and homozygous mutants and wild‐type littermates.

**Result:**

Barnes Maze Acquisition was performed on males (*N* = 4‐5/genotype) after habituation and several days training. Heatmaps of grouped averages for acquisition day 5 show that *ErbB4‐R927Q* mutants (homozygotes > heterozygotes) take longer finding the goal box, suggesting a gene dosage‐dependent defect in spatial learning. CatWalk‐XT was performed on *Erbb4‐R927Q* mutant females (*N* = 4‐6/genotype), with six complaint post‐training walks/animal. Significantly lower mean intensities of the most intense paw prints occurred in the hindlimbs of mutants (Tukey's 2‐way ANOVA). Multiple gene‐dosage‐dependent trends in altered gait metrics occurred, including footfall patterns, 2D/3D mean/max intensities, footfall sequence, print positions, standing and diagonal mean phase dispersions. DXA scans on *Erbb4‐R927Q* mutant and wild‐type males (*N* = 5‐9 per genotype) showed decreased body weights and fat percentages in homozygotes. Whole body and limb regions of interest showed decreases in bone mineral density and content that was greater in homozygotes than in heterozygotes.

**Conclusion:**

Initial findings suggest gene dosage‐dependent abnormalities in *Erbb4‐R927Q* mutants that have features of FTLD and ALS. A wider battery of tests on more animals, at different ages and including the *Erbb4‐I712M* mutants are underway. Neuropathology is being assessed following consensus recommendations for FTLD and ALS. Stereology/morphometry and other methods will assess loss of *ErbB4*‐expressing inhibitory interneurons and synaptic loss and how this evolves temporally.

